# The Relationship Between Balance Measured With a Modified Bathroom Scale and Falls and Disability in Older Adults: A 6-Month Follow-Up Study

**DOI:** 10.2196/jmir.3802

**Published:** 2015-05-27

**Authors:** Joan Vermeulen, Jacques CL Neyens, Marieke D Spreeuwenberg, Erik van Rossum, April BCG Boessen, Walther Sipers, Luc P de Witte

**Affiliations:** ^1^School for Public Health and Primary Care (CAPHRI)Department of Health Services ResearchMaastricht UniversityMaastrichtNetherlands; ^2^Research Center Technology and CareFaculty of HealthZuyd University of Applied SciencesHeerlenNetherlands; ^3^Expertise Center for Elderly CareOrbis Medical CenterSittardNetherlands

**Keywords:** telemonitoring, balance, bathroom scale, older adults, falls, disability, validity

## Abstract

**Background:**

There are indications that older adults who suffer from poor balance have an increased risk for adverse health outcomes, such as falls and disability. Monitoring the development of balance over time enables early detection of balance decline, which can identify older adults who could benefit from interventions aimed at prevention of these adverse outcomes. An innovative and easy-to-use device that can be used by older adults for home-based monitoring of balance is a modified bathroom scale.

**Objective:**

The objective of this paper is to study the relationship between balance scores obtained with a modified bathroom scale and falls and disability in a sample of older adults.

**Methods:**

For this 6-month follow-up study, participants were recruited via physiotherapists working in a nursing home, geriatricians, exercise classes, and at an event about health for older adults. Inclusion criteria were being aged 65 years or older, being able to stand on a bathroom scale independently, and able to provide informed consent. A total of 41 nursing home patients and 139 community-dwelling older adults stepped onto the modified bathroom scale three consecutive times at baseline to measure their balance. Their mean balance scores on a scale from 0 to 16 were calculated—higher scores indicated better balance. Questionnaires were used to study falls and disability at baseline and after 6 months of follow-up. The cross-sectional relationship between balance and falls and disability at baseline was studied using t tests and Spearman rank correlations. Univariate and multivariate logistic regression analyses were conducted to study the relationship between balance measured at baseline and falls and disability development after 6 months of follow-up.

**Results:**

A total of 128 participants with complete datasets—25.8% (33/128) male—and a mean age of 75.33 years (SD 6.26) were included in the analyses of this study. Balance scores of participants who reported at baseline that they had fallen at least once in the past 6 months were lower compared to nonfallers—8.9 and 11.2, respectively (*P*<.001). The correlation between mean balance score and disability sum-score at baseline was -.51 (*P*<.001). No significant associations were found between balance at baseline and falls after 6 months of follow-up. Baseline balance scores were significantly associated with the development of disability after 6 months of follow-up in the univariate analysis—odds ratio (OR) 0.86 (95% CI 0.76-0.98)—but not in the multivariate analysis when correcting for age, gender, baseline disability, and falls at follow-up—OR 0.94 (95% CI 0.79-1.11).

**Conclusions:**

There is a cross-sectional relationship between balance measured by a modified bathroom scale and falls and disability in older adults. Despite this cross-sectional relationship, longitudinal data showed that balance scores have no predictive value for falls and might only have limited predictive value for disability development after 6 months of follow-up.

##  Introduction

There are indications that older adults who suffer from poor balance have an increased risk for adverse health outcomes, such as falls, mobility-related disability, and disability in daily activities [1-4]. Monitoring the development of balance over time enables early detection of balance decline. Providing interventions aimed at improving balance and preventing falls or disability could be beneficial to older adults with decreased balance because it can reduce their risk of these adverse outcomes [5-8].

Possibilities for monitoring the development of balance over time in older adults are clinical balance tests that are conducted by care professionals [9-11], (expensive) force plate equipment that is available in clinical/laboratory settings [12], and innovative telemonitoring devices [13-16]. The latter can be used by older adults in their own homes without the presence of a care professional. This can facilitate regular monitoring and early detection of change over time. Furthermore, such telemonitoring devices can provide direct information regarding balance changes to the user, which can support self-management.

A telemonitoring device appropriate for home-based self-monitoring of balance is a modified bathroom scale [13]. This device uses an algorithm to calculate balance parameters and is equipped with Bluetooth, which enables the transfer of balance and weight data to a mobile phone-based app. Via the app, older adults can receive information about their own balance scores, or changes in these scores. Furthermore, the data could be forwarded to a database that can be accessed by care professionals, which enables them to monitor the development of balance in their patients over time from a distance [17,18]. Older adults are able to use the modified bathroom scale for home-based self-monitoring of balance because it does not differ from a normal bathroom scale [13,19]. Previous research that compared balance scores of the modified bathroom scale to clinical balance tests, such as the Performance-Oriented Mobility Assessment or Timed Up and Go, suggests good construct validity, especially in older adults with slightly diminished balance [20]. Besides that, the modified bathroom scale provides estimates of balance-related parameters similar to a force plate [21].

Since the bathroom scale seems to be able to provide valid balance measurements, it can be used to identify balance decline in older adults. However, no studies have been conducted yet in which the predictive validity of balance scores of the modified bathroom scale on adverse outcomes has been studied. Information regarding predictive validity can help older adults and care professionals to interpret the balance scores. Furthermore, it is important to know whether lower balance scores are associated with adverse outcomes in order to decide whether, or which, preventive interventions would be justified when balance decline is detected. Therefore, the aim of this study is to explore the relationship between balance scores of the modified bathroom scale and falls and disability in older adults.

## Methods

### Design, Setting, and Participants

A longitudinal study with 6-month follow-up was conducted in two Southern provinces of the Netherlands between October 2012 and July 2013. Participants were recruited via different settings to ensure that older adults with different balance levels, ranging from very poor to very good, were represented in the study sample. Participants were recruited via physiotherapists working in two nursing homes, the outpatient clinic of a geriatrician, exercise classes for older adults, and at an event about health for older adults. To be eligible for inclusion, participants had to be aged above 65 years, able to stand on the bathroom scale independently, and able to provide written informed consent.

Potential participants who met the eligibility criteria mentioned above received an invitation letter from their physiotherapist (n=48), geriatrician (n=28), exercise instructor (n=72), or the researcher (n=60) that contained information regarding the study. Invitations were handed out during regular physiotherapy sessions, consultations with the geriatrician, exercise classes, and at an event about health for older adults. Before handing out the invitations, the physiotherapists, geriatricians, exercise instructor, and researcher checked whether a person was able to provide written informed consent. Those who met the eligibility criteria and signed informed consent documents were included in the study. Once written informed consent was provided, participants measured their balance using the modified bathroom scale and filled out a paper-based questionnaire. After 6 months of follow-up, the same questionnaire was sent to the participants. Nonresponders received a reminder after 3 weeks asking them to return the questionnaire. This study was approved by the Medical Ethical Committee Atrium-Orbis-Zuyd (Reference: NL 142245709, July 23, 2012).

### Measurements

Participants conducted balance measurements at baseline and filled out a questionnaire regarding participant characteristics (ie, age, gender, chronic conditions, psychotropic drug use), falls, and disability at baseline and at the 6-month follow-up measurement.

**Figure 1:**
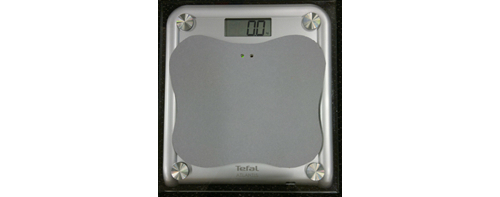
Modified bathroom scale.

Balance was measured using the modified bathroom scale (see Figure 1). The scale is equipped with an infrared sensor at the front which activates the bathroom scale. All participants were instructed to stand in front of the bathroom scale and to step onto it when the number “0.0” appeared on the display. They were instructed to step down backwards once their weight appeared on the screen. The modified bathroom scale uses the signals from four pressure sensors located in the corners of the scale to collect information regarding two dynamic and two static balance parameters. An overall balance score is calculated using the information regarding the following four parameters: step on delay, rise rate, surface under the stabilogram, and average velocity of the trajectory. Each parameter is scored on a scale from 0 to 4 which results in an overall balance score between 0 and 16—a higher score indicates better balance. Detailed information regarding the parameters and the calculation of the overall balance score is described by Duchêne and Hewson [13]. Participants stepped onto the bathroom scale three consecutive times which resulted in three balance scores. The mean balance score of these three measurements was calculated and used in the analyses. The researchers were present when participants used the bathroom scale to provide help and instructions when needed.

Falls were defined as unintentionally coming to rest on the ground, floor, or other lower level. Via the questionnaire, participants were asked to report whether they had fallen in the past 6 months. Those who had fallen at least once in the past 6 months were considered fallers.

Disability was measured using the Groningen Activity Restriction Scale (GARS), which is a valid and reliable measuring instrument [22]. The GARS consists of 18 items, 11 of which refer to activities of daily living (ADL) and seven of which refer to instrumental activities of daily living (IADL). A copy of the GARS is provided in Multimedia Appendix 1. For each item, participants indicated on a 4-point scale whether they could perform the activity independently without any difficulty (score of 1), independently with some difficulty (score of 2), independently with great difficulty (score of 3), or whether they could not execute the activity independently (score of 4). So, if participants scored 4, they depended on other people for the performance of that activity. Overall disability, ADL disability, and IADL disability sum-scores were calculated and ranged from 18 to 72, 11 to 44, and 7 to 28, respectively—higher scores indicated higher disability levels. Disability development after 6 months of follow-up was operationalized as increased dependence in daily activities—ADL and IADL combined—meaning that a participant was dependent in at least one more activity of the GARS at follow-up compared to baseline. This was calculated by subtracting the number of activities in which a participant was dependent at baseline from the number of activities in which a participant was dependent at follow-up.

### Statistical Analyses

Descriptive statistics were used to provide information regarding the baseline characteristics of the participants. Categorical variables were expressed with percentages and continuous variables with means and standard deviations.

To study the reliability of the modified bathroom scale, intraclass correlation coefficients (ICCs)—two-way random effects using absolute agreement—of the balance scores were calculated. ICCs were calculated for the three repeated balance scores and separately for each of the four balance parameters—step on delay, rise rate, surface under the stabilogram, and average velocity of the trajectory.

The independent samples *t* test was conducted to determine whether participants who reported at baseline that they had fallen at least once in the past 6 months had a lower mean balance score compared to participants who had not fallen in the 6 months before baseline. To study the relationship between balance and disability at baseline, Spearman rank correlations between the mean balance score and overall, ADL, and IADL disability sum-scores at baseline were calculated.

To study the relationship between balance scores at baseline and falls and disability after 6 months of follow-up, univariate and multivariate logistic regression analyses were conducted. Six univariate regression analyses were conducted with baseline balance scores of the modified bathroom scale, faller at baseline (1=yes, 0=no), baseline disability (ie, GARS overall sum-score), psychotropic drug use at baseline (1=yes, 0=no), gender (1=female, 0=male), and age as independent variables and faller after 6 months of follow-up as dependent variable. Five univariate regression analyses were conducted with baseline balance scores of the modified bathroom scale, baseline disability (ie, GARS overall sum-score), faller at follow-up (1=yes, 0 = no), gender, and age as independent variables and disability development after 6 months of follow-up as dependent variable. In addition, two multivariate logistic regression analyses were conducted to study the predictive value of the bathroom scale balance score at baseline on falling and development of disability after 6 months of follow-up, while correcting for relevant baseline variables—age, gender, faller at baseline, use of psychotropic drugs, GARS sum-score at baseline—and faller at follow-up.

Disability development after 6 months of follow-up was the primary outcome of our study. According to Green, at least 90 participants should be included in the multivariate analysis with five independent variables to ensure sufficient power [23]. To ensure that enough participants could be included in the analyses, 180 participants were recruited at baseline, taking into account a “worst-case scenario” of not being able to include 50% of the participants in the final analyses due to loss to follow-up or incomplete datasets. All analyses were conducted using SPSS version 19.0 (SPSS Inc, IBM Corp, Armoch, NY). Assumptions for the *t* tests and logistic regression models were checked and met.

## Results

### Participants

**Figure 2:**
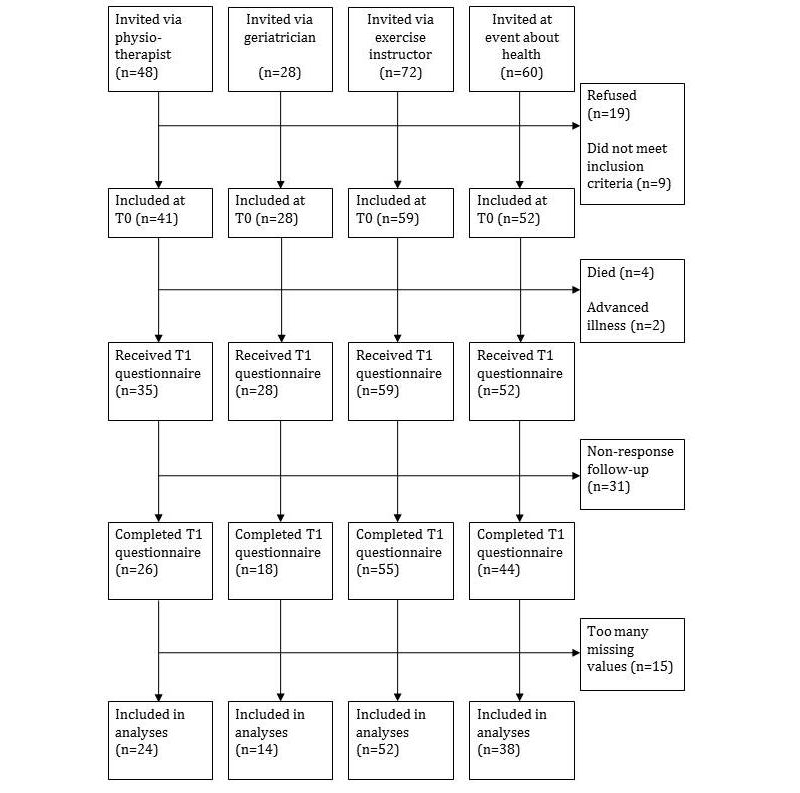
Flowchart of participants. Baseline (T0), 6-month follow-up (T1).

Figure 2 provides an overview of the inclusion process. A total of 208 participants received an invitation and 180 provided written informed consent and participated in the baseline measurement. After the 6-month follow-up, 4 participants had died and 2 could not be approached for the follow-up measurement due to advanced illness. Of the 174 participants who received the 6-month follow-up questionnaire, 143 returned it (82.2%). Finally, 15 participants were excluded from the analyses because they had four or more missing values on the GARS, or because they had not answered the question regarding falls. This resulted in a study sample of 128 participants with complete datasets—25.8% (33/128) male—and a mean age of 75.33 years (SD 6.26). More information regarding the baseline characteristics of the study sample is provided in Table 1.

**Table 1:** Baseline characteristics of participants (n=128).

Characteristics		Mean (SD) or n (%)
Age in years, mean (SD)		75.33 (6.26)
**Gender, n (%)**		
	Male	33 (25.8)
	Female	95 (74.2)
**Chronic diseases, n (%)**		
	Diabetes	21 (16.4)
	COPD^a^/asthma	7 (5.5)
	Cardiovascular diseases	39 (30.5)
	Arthritis	36 (28.1)
	Parkinson’s disease/MS^b^	7 (5.5)
Balance score, mean (SD)		10.63 (3.17)
Falls in past 6 months, n (%)		31 (24.2)
**Disability sum-scores, mean (SD)**		
	GARS^c^ overall sum-score (ADL^d^ + IADL^e^)	27.42 (12.00)
	GARS ADL sum-score	15.13 (5.96)
	GARS IADL sum-score	12.29 (6.61)
**Dependence, n (%)**		
	Dependent in one activity	20 (15.6)
	Dependent in two activities	12 (9.4)
	Dependent in three activities	10 (7.8)
	Dependent in four activities	25 (19.5)

^a^Chronic obstructive pulmonary disease (COPD).

^b^Multiple sclerosis (MS).

^c^Groningen Activity Restriction Scale (GARS), range 18 to 72.

^d^Activities of daily living (ADL), range 11 to 44.

^e^Instrumental activities of daily living (IADL), range 7 to 28.

Out of 128 participants, 23 (18.0%) reported that they had fallen at least once during the follow-up period of the study. Of these 23 participants, 15 (65%) also reported a fall during the 6 months before the baseline measurement. After 6 months of follow-up, the level of dependence increased in 32 of the 128 participants (25.0%), meaning that these participants needed help from another person with at least one more activity compared to baseline.

There were no demographical differences between participants who completed the study and those who were lost to follow-up. The mean balance scores of participants who dropped out of the study after the baseline measurement were significantly lower, namely 9.26 (SD 3.69), compared to those of participants who were included in the analyses, namely 10.63 (SD 3.17) (*P*=.009). Furthermore, baseline IADL disability sum-scores were higher in the group of participants who were not included in the analyses of this study, namely 14.52 (SD 7.35), compared to those who were included in the study sample, namely 12.29 (SD 6.61) (*P*=.049). Dropout was highest among participants who were recruited via the physiotherapists working in a nursing home and via the geriatrician.

### Reliability

The ICC for three consecutive balance scores of the modified bathroom scale was .70 (95% CI .62-.77). The ICCs for the four separate balance parameters—step on delay, rise rate, surface under the stabilogram, and average velocity of the trajectory—were .31 (95% CI .20-.43), .72 (95% CI .64-.79), .54 (95% CI .43-.63), and .54 (95% CI .44-.64), respectively.

### Cross-Sectional Relationship Between Balance, Falls, and Disability

Balance scores of participants who had fallen at least once in the past 6 months before baseline were lower compared to nonfallers—8.9 and 11.2, respectively (*P*<.001, 95% CI 1.08-3.54). Correlations between mean balance score and overall, ADL, and IADL disability sum-scores at baseline were -.51, -.42, and -.46, respectively (*P*<.001).

### Relationship Between Baseline Balance and Falls and Disability at Follow-Up

Results of the univariate regression analyses are presented in Table 2 and results of the multivariate regression analyses are presented in Table 3. Falls reported at baseline were significantly associated with falls during the 6-month follow-up in the univariate analyses—odds ratio (OR) 10.43 (95% CI 3.80-28.63)—and in the multivariate analysis—OR 14.02 (95% CI 4.06-48.50). Disability sum-score at baseline was also significantly associated with falls during the 6-month follow-up in the univariate analysis—OR 1.04 (95% CI 1.00-1.07)—but not in the multivariate analysis. Baseline balance scores were significantly associated with the development of disability after the 6-month follow-up in the univariate analyses—OR 0.86 (95% CI 0.76-0.98). Furthermore, disability level at baseline was significantly associated with disability development after 6 months of follow-up in the univariate analyses—OR 1.04 (95% CI 1.00-1.07). None of the variables entered into the multivariate regression model was predictive of disability development after 6 months of follow-up.

**Table 2:** Univariate association between baseline variables and falls and disability development after 6 months of follow-up (n=128).

Independent variable	Dependent variable			
	Falls at follow-up, OR^a^ (95% CI)	*P*	Disability development at follow-up, OR (95% CI)	*P*
Mean balance score at baseline (scale 1 to 16)	0.96 (0.84-1.11)	.62	0.86 (0.76-0.98)	.03^b^
Age in years	1.00 (0.93-1.07)	.95	1.07 (1.00-1.14)	.05
Gender (0=male, 1=female)	0.59 (0.22-1.54)	.28	0.47 (0.20-1.11)	.08
Faller at baseline (0=no, 1=yes)	10.43 (3.80-28.63)	<.001	N/A^c^	N/A
Psychotropic drugs at baseline (0=no, 1=yes)	2.50 (0.76-8.19)	.13	N/A	N/A
GARS^d^ sum-score at baseline (scale 18 to 72)	1.04 (1.00-1.07)	.03	1.04 (1.00-1.07)	*.03*
Faller at follow-up (0=no, 1=yes)	N/A	N/A	2.29 (0.88-5.97)	.09

^a^Odds ratio (OR).

^b^Significant associations (univariate) are shown in italics.

^c^Not applicable (N/A): variable not entered in analysis.

^d^Groningen Activity Restriction Scale (GARS).

**Table 3:** Multivariate predictors of falls and disability development after 6 months of follow-up (n=128).

Independent variable	Dependent variable			
	Falls at follow-up^a^, OR^b^ (95% CI)	*P*	Overall disability at follow-up^a^, OR (95% CI)	*P*
Mean balance score at baseline (scale 1 to 16)	1.21 (0.95-1.57)	.13	0.94 (0.79-1.11)	.45
Age in years	1.00 (0.90-1.10)	.92	1.04 (0.97-1.12)	.29
Gender (0=male, 1=female)	0.51 (0.16-1.60)	.25	0.50 (0.20-1.24)	.13
Faller at baseline (0=no, 1=yes)	14.02 (4.06-48.50)	<.001 ^*c*^	N/A^d^	N/A
Psychotropic drugs at baseline (0=no, 1=yes)	0.86 (0.18-4.28)	.86	N/A	N/A
GARS^e^ sum-score at baseline (scale 18 to 72)	1.04 (0.98-1.09)	.17	1.02 (0.98-1.06)	.34
Faller at follow-up (0=no, 1=yes)	N/A	N/A	2.00 (0.72-5.54)	.19

^a^Adjusted *R* ^2^ for falls at follow-up was 0.30, adjusted *R* ^2^ for overall disability at follow-up was 0.11.

^b^Odds ratio (OR).

^c^Significant association (multivariate) is shown in italics.

^d^Not applicable (N/A): variable not entered in model.

^e^Groningen Activity Restriction Scale (GARS).

## Discussion

### Principal Findings and Comparison to Previous Research

The results of this study indicate that the reliability of the balance scores of the modified bathroom scale is acceptable since, according to Nunnally, ICCs at or above .70 are considered acceptable [24,25]. There seems to be a significant cross-sectional relationship between balance scores and falls since the group of participants who suffered a fall in the past 6 months before baseline had significantly lower balance scores compared to those who did not fall. The difference between these groups was 2.3 points on a scale from 0 to 16. Furthermore, there was a significant and substantial correlation between balance scores and disability sum-scores at baseline, which revealed that poorer balance was associated with higher disability levels. Despite this cross-sectional relationship, longitudinal data showed that balance scores have no predictive value for falls in the next 6 months, and maybe only limited predictive value for disability development after 6 months of follow-up. No significant relationship was found between balance at baseline and falls after 6 months of follow-up in the univariate and multivariate regression analyses. Baseline balance score was associated with disability development in the univariate regression analyses, which indicated that older adults with poorer balance had a higher risk of developing disability after 6 months of follow-up. However, when correcting for age, gender, and baseline disability in the multivariate regression analyses, this association was no longer significant.

Previous studies have been conducted regarding the relationship between balance measured by clinical balance tests, force plates, or telemonitoring devices and falls and disability in older adults. Most studies focused on the predictive validity of clinical balance tests and these studies suggested that poor balance predicts a moderately increased risk of these adverse outcomes in older adults [1-3,26]. Previous studies regarding the predictive value of balance-related parameters measured with a force plate on falls revealed contradictory results—some studies reported that force plate measurements predicted falls, whereas other studies reported that no associations were found [27,28]. Previous research regarding innovative telemonitoring technologies that can be used for home-based self-monitoring of balance mostly concerns the Nintendo Wii. A recent review regarding the use of the Nintendo Wii for the assessment and training of balance revealed that, despite the fact that the Wii Balance Board can be used as a proxy for measurements conducted with a force plate, its software is not very effective in determining balance status [29]. Furthermore, correlations between balance scores of the Wii Balance Board and clinical balance tests are low [30,31]. No previous studies have been conducted yet regarding the predictive value of the Wii Balance Board, or other home-based balance telemonitoring devices, on adverse health outcomes in older adults. The results of our study were in line with previous research that indicated that falls in the past are a strong predictor of falls in the future [32,33].

This study only partly confirmed findings from previous research, since it revealed a cross-sectional relationship between balance and falls and disability, but no association between balance scores at baseline and falls and disability development after 6 months of follow-up could be demonstrated. A possible explanation for this could be that the follow-up period of this study was short compared to other studies that focused on the predictive validity of clinical balance tests on falls and disability [34]. Due to this shorter follow-up period, not many falls or changes in the level of dependence occurred in this study. In addition, previous research suggests that balance is not always a very strong predictor of future falls and disability development, which could explain why no significant relationship was found when correcting for other relevant baseline characteristics. Another possible explanation of why studies focusing on clinical balance tests revealed moderate predictive value of balance in older adults, and why scores of the modified bathroom scale were not predictive of future falls and disability in this study, could be that professionals who conduct such clinical balance tests often take into account different aspects of balance, or physical functioning, and have their clinical expertise to rely on when estimating the risk for falls or disability development.

### Strengths and Limitations

In total, 128 participants, which is 71.1% of the baseline study sample of 180, could be included in the analyses of this study. Dichotomization of dependent variables could have negatively influenced statistical power. The follow-up period of 6 months is relatively short compared to previous research regarding the predictive validity of balance in older adults. Possibly, as a result of the shorter follow-up period, only 23 out of 128 participants (18.0%) reported that they had suffered one or multiple falls during the study. No distinction was made in the analyses between participants who fell once during the follow-up period and those who fell multiple times because the groups would become even smaller, which would negatively influence the statistical power. The relatively short follow-up period of our study is not necessarily a limitation since, for the early identification of older adults with balance decline who could benefit from preventive intervention programs, it is more useful to know the short-term predictive value of the balance scores of the modified bathroom scale. It makes more sense to start with a preventive intervention when short-term predictors are present in older adults compared to a situation in which it will take a few years before adverse outcomes will develop.

The number of participants who reported increased overall disability after 6 months of follow-up was higher, namely 32 out of 128 (25.0%), compared to the number of participants who reported a fall. However, a possible limitation of this study could be that participants who were lost to follow-up reported higher disability levels and lower balance scores at baseline compared to those who remained in the sample. This may have influenced the results of our study because the disability levels at follow-up and the variation in scores on dependent and independent variables might have been higher if those participants could have been included in the analyses. Based on the available data, no firm conclusions can be drawn regarding the extent to which this selective loss to follow-up has influenced the results of our study.

It should be noted that all balance measurements were performed under the supervision of a researcher. This can yield different results compared to home-based measurements using the modified bathroom scale. Balance scores of the modified bathroom scale could have been higher during this study because participants might be more alert, step onto the bathroom scale quicker, or try to stand very still when the researcher is present, whereas they might not do this when performing home-based measurements alone. Based on this study, no estimates can be provided on how the reliability of balance scores of the modified bathroom scale are influenced by the setting in which they are conducted (ie, research setting vs home-based setting), and to what extent the setting might influence the relationship with falls and disability. The ICCs that were calculated to evaluate test-retest reliability of the four separate balance parameters revealed that three out of four were not adequate, taking into account the proposed cutoff point of .70 for minimum reliability by Nunnally [25]. The ICC of step on delay was lowest (.31), followed by surface under the stabilogram and average velocity of the trajectory (both .54). This means that the scores of these parameters differed considerably across the three consecutive measurements of a participant. To what extent these parameters, and thereby the balance scores, are influenced by the participant "learning" how to step onto the bathroom scale, presence of the researcher, cognitive functioning, or other factors cannot be concluded based on this study.

### Conclusions

There is a cross-sectional relationship between balance measured with a modified bathroom scale and falls and disability in older adults. Longitudinal data did not confirm this, which suggests that balance scores of the modified bathroom scale have no predictive value for falls and might have only limited predictive value for disability development after 6 months of follow-up. Research with a larger sample and longer follow-up period is needed to confirm or contradict these findings, and to determine whether balance score cutoff points can be formulated, for different subpopulations, that identify older adults with increased risk for adverse health outcomes. Follow-up studies in which older adults use the bathroom scale on a regular basis (eg, daily or weekly) for home-based monitoring of balance would provide useful information regarding the variation in balance scores among older adults and regarding clinically relevant changes. Such information is needed before the bathroom scale can be implemented in practice.

## Multimedia Appendix 1

Groningen Activity Restriction Scale (GARS).

## Abbreviations

ADL: activities of daily living
COPD: chronic obstructive pulmonary disease
GARS: Groningen Activity Restriction Scale
IADL: instrumental activities of daily living
ICC: intraclass correlation coefficient
MS: multiple sclerosis
N/A: not applicable
OR: odds ratio
T0: baseline
T1: 6-month follow-up
