# Supplementary material for: The Relationship Between Balance Measured With a Modified Bathroom Scale and Falls and Disability in Older Adults: A 6-Month Follow-Up Study
Source: J Med Internet Res. 2015 May 27;17(5):e131. doi: 10.2196/jmir.3802 (PMC4468574; doi:10.2196/jmir.3802)
Supplement: Supplementary file 1 [file jmir_v17i5e131_app1.pdf]

Appendix 1: Groningen Activity Restriction Scale (GARS)

The following questions refer to daily activities which should be performed frequently. In each question it is asked whether you *are able to perform* the activity at this moment. It is not intended to assess whether you are actually performing the activities, but if you can do them if necessary.

Response categories for each item

1. Yes, I can do it fully independently without any difficulty
2. Yes, I can do it fully independently but with some difficulty
3. Yes, I can do it fully independently but with great difficulty
4. No, I cannot do it fully independently, I can only do it with someone's help

GARS items

1. Can you, fully independently, dress yourself?
2. Can you, fully independently, get in and out of bed?
3. Can you, fully independently, stand up from sitting in a chair?
4. Can you, fully independently, wash your face and hands?
5. Can you, fully independently, wash and dry your whole body?
6. Can you, fully independently, get on and off the toilet?
7. Can you, fully independently, feed yourself?
8. Can you, fully independently, get around in the house (if necessary with a cane or walker)?
9. Can you, fully independently, go up and down the stairs?
10. Can you, fully independently, walk outdoors (if necessary with a cane or walker)?
11. Can you, fully independently, take care of your feet and toenails?
12. Can you, fully independently, prepare breakfast or lunch?
13. Can you, fully independently, prepare dinner?
14. Can you, fully independently, do "light" household activities (for example, dusting and tidying up)?
15. Can you, fully independently, do "heavy" household activities (for example mopping, cleaning the windows, and vacuuming)?
16. Can you, fully independently, wash and iron your clothes?
17. Can you, fully independently, make the beds?
18. Can you, fully independently, do the shopping?
